# Identifying metabotypes of insulin resistance severity in children with metabolic syndrome

**DOI:** 10.1186/s12933-024-02412-x

**Published:** 2024-08-27

**Authors:** Álvaro González-Domínguez, Jesús Domínguez-Riscart, Otto Savolainen, Alfonso Lechuga-Sancho, Rikard Landberg, Raúl González-Domínguez

**Affiliations:** 1 Instituto de Investigación e Innovación Biomédica de Cádiz (INiBICA), Hospital Universitario Puerta del Mar, Universidad de Cádiz, Cádiz, 11009 Spain; 2 Division of Liver Diseases, Icahn School of Medicine at Mount Sinai, New York, 10029 USA; 3 Unidad de Endocrinología Pediátrica y Diabetes, Servicio de Pediatría, Hospital Universitario Puerta del Mar, Cádiz, 11009 Spain; 4 Division of Food and Nutrition Science, Department of Life Sciences, Chalmers University of Technology, Gothenburg, SE-412 96 Sweden; 5 Departamento Materno Infantil y Radiología, Facultad de Medicina, Universidad de Cádiz, Cádiz, 11009 Spain

**Keywords:** Insulin resistance, Childhood obesity, Metabolomics, Metabolic flexibility

## Abstract

**Background:**

Insulin resistance is a frequent precursor of typical obesity and metabolic syndrome complications. However, accurate diagnosis remains elusive because of its pathophysiological complexity and heterogeneity. Herein, we have explored the utility of insulin secretion dynamics in response to an oral glucose tolerance test as a surrogate marker to identify distinct metabotypes of disease severity.

**Methods:**

The study population consisted of children with obesity and insulin resistance, stratified according to the post-challenge insulin peak timing (i.e., early, middle, and late peak), from whom fasting and postprandial plasma and erythrocytes were collected for metabolomics analysis.

**Results:**

Children with late insulin peak manifested worse cardiometabolic health (i.e., higher blood pressure, glycemia, and HOMA-IR scores) than early responders. These subjects also showed more pronounced changes in metabolites mirroring failures in energy homeostasis, oxidative stress, metabolism of cholesterol and phospholipids, and adherence to unhealthy dietary habits. Furthermore, delayed insulin peak was associated with impaired metabolic flexibility, as reflected in compromised capacity to regulate mitochondrial energy pathways and the antioxidant defense in response to glucose overload.

**Conclusions:**

Altogether, these findings suggest that insulin resistance could encompass several phenotypic subtypes characterized by graded disturbances in distinctive metabolic derangements occurring in childhood obesity, which serve as severity predictive markers.

**Supplementary Information:**

The online version contains supplementary material available at 10.1186/s12933-024-02412-x.

## Background

Insulin resistance (IR) is a clinical condition characterized by impaired response of target tissues to insulin. This disorder causes disruptions in carbohydrate disposal and, hence, results in compensatory upregulation of pancreatic β-cell function to maintain a tight control of blood glucose levels, ultimately leading to chronic hyperinsulinemia [1]. Although its etiology is multifactorial, involving genetic determinants (e.g., mutations in insulin receptors and signaling proteins), lifestyle (e.g., nutritional imbalances, sedentarism, medication use), and other susceptibility factors (e.g., puberty, ethnicity), excess body fat and related pathogenic events (e.g., inflammation, lipotoxicity) have been identified as the primary triggers of this disorder. In this respect, it is well-recognized that IR is the most prevalent comorbidity of childhood obesity, frequently participating in the onset of other complications [2]. Thus, IR stands out as a cornerstone in the development of a constellation of cardiometabolic disturbances (e.g., dyslipidemia, hypertension) that constitute the so-called metabolic syndrome, which is in turn associated with increased risk for type 2 diabetes mellitus and cardiovascular diseases during adulthood. However, despite its clinical significance, accurate IR assessment in children remains elusive [1]. The hyperinsulinemic euglycemic clamp is the gold standard for diagnosing IR, but this is a time-consuming, labor-intensive, invasive, and expensive technique, so it is rarely applied in clinical practice. As an alternative, other indirect methods have been proposed based on fasting blood determinations (e.g., the homeostatic model assessment of IR, HOMA-IR; the quantitative insulin sensitivity check index; QUICKI), as well as by means of evaluating changes in carbohydrate metabolism in response to an oral glucose tolerance test (OGTT). Nonetheless, these surrogate markers have been reported to suffer from lower accuracy, higher interindividual variability, limited ability to predict the risk of future complications, and lack of international consensus on reference values, especially in children, which altogether has precluded their routine implementation [3]. Consequently, there is an urgent need to define novel markers to better understand IR pathophysiology and improve prediction of adverse outcomes.

The OGTT is a challenge test frequently employed to assess the individuals’ capacity to handle a glycemic load and, therefore, to diagnose disorders related to carbohydrate metabolism. Unlike fasting measurements, which are expected to be tightly regulated by homeostasis, the postprandial adaptation to transient nutritional stressors (i.e., metabolic flexibility) has been proposed as a more reliable indicator of metabolic health [4]. In this sense, the determination of blood glucose and insulin levels along the OGTT has long been used for evaluating IR through various indices (e.g., the whole-body insulin sensitivity index, WBISI), but growing evidence suggests that the shapes of these post-challenge curves could represent a more valuable measure to assess IR severity. In a pioneer work conducted in 2013, Hayashi et al. described that a late insulin peak during an OGTT is associated with exacerbated IR and higher risk of developing type 2 diabetes when compared to early responders [5]. Although scarce data is available in pediatric populations, recent findings pinpoint that delayed insulin response in children with obesity is mirrored in worsen metabolic profiles (e.g., higher HOMA-IR scores and triglyceride content, lower HDL cholesterol) [6], oxidative stress and inflammasome activation [7], as well as altered metal homeostasis [8]. In this context, further research is crucial to decipher the molecular disturbances that altered patterns of insulin secretion imprint in children with IR, which may facilitate its diagnosis and severity stratification. For this purpose, metabolomics has proven to be a powerful tool to unravel the influence of interindividual variability factors and, thus, to identify distinct phenotypic subtypes (i.e., metabotypes) behind complex disorders [9].

Herein, we have applied state-of-the-art metabolomics to plasma and erythrocytes from a population encompassing children with obesity and IR, who were subjected to an OGTT and further stratified according to their insulin curve morphology: children showing an early insulin peak (t = 30 min), middle insulin peak (t = 60 min), and late insulin peak (t ≥ 90 min). Then, biological samples were collected at both fasting and along the OGTT to investigate the association between postprandial insulin dynamics, IR-related metabolic impairments, and individuals’ metabolic flexibility.

## Methods

### Study design

The study population consisted of 76 children, aged 6 to 16 years (48 male, 28 female), with obesity and IR who were recruited at “Hospital Universitario Puerta del Mar” (Cádiz, Spain). The inclusion criteria were having a body mass index (BMI) over two standard deviations above the age/sex-adjusted mean of the Spanish reference population, and fulfilling at least one of the following IR hallmarks: (i) HOMA-IR score above 3.5, (ii) fasting insulin levels above 15 µU/mL, (iii) insulin levels above 75 µU/mL at 120 min of the OGTT, (iv) insulin levels above 150 µU/mL at any time point of the OGTT [10]. Then, participants were stratified according to the OGTT-induced insulin profile as reported elsewhere [6]: children showing an early insulin peak (t = 30 min, *N* = 18), middle insulin peak (t = 60 min, *N* = 19), and late insulin peak (t ≥ 90 min, *N* = 39). Subjects with other known chronic systemic diseases or suffering of acute infectious processes were not eligible for the study. Pediatric endocrinologists registered anthropometric characteristics of the study population (i.e., weight, height, BMI), and the updated version of the KIDMED questionnaire was employed to assess dietary habits [11]. Venous blood samples were collected in the morning after overnight fasting, as well as along the OGTT (i.e., 30, 60, 90, and 120 min), using BD Vacutainer EDTA tubes. Then, blood tubes were centrifuged at 1500 g for 10 min at 4 °C to separate the plasma, and cell pellets were subjected to three cycles of washing with cold saline solution (9 g/L NaCl, 4 °C) and subsequent centrifugation (1500 g, 5 min, 4 °C) to recover erythrocytes. A plasma aliquot was employed to determine glucose and insulin concentrations using an Alinity automatic analyzer (Abbot, Madrid, Spain). The HOMA-IR score was calculated by applying the formula: HOMA-IR = (Glc × Ins) × 0.055/22.5, where Glc and Ins refer to fasting glucose and insulin concentrations, expressed as mg/dL and µU/mL, respectively. The rest of samples were stored at -80 °C until metabolomics analysis. The study was performed in accordance with the principles contained in the Declaration of Helsinki. The Ethical Committee of Hospital Universitario Puerta del Mar (Cádiz, Spain) approved the study protocol (Ref. PI22/01899), and all participants and/or legal guardians provided written informed consent.

### Metabolomics workflow

Plasma and erythrocyte samples collected at fasting and along the OGTT (i.e., 60 and 120 min) were subjected to a metabolomics workflow comprising mass spectrometry-based analysis [12], raw data preprocessing using MS-DIAL software [13], and quality control assessment according to the QC*omics* recommendations [14], as detailed in previous publications. Then, metabolites of interest were annotated following the guidelines reported by the Metabolomics Standards Initiative (MSI), based on matching experimental data (i.e., accurate m/z and tandem spectra, maximum error mass: 10 ppm) against those available in databases (i.e., Human Metabolome Database, METLIN), and subsequent analysis of pure standards when available [15]. Complementarily, phospholipids and phase II metabolites were identified thanks to their characteristic fragment spectra [16, 17].

### Statistical analysis

Using a statistical pipeline well-established among the metabolomics community [18], data were subjected to multivariate and univariate tools to identify differential metabolites between the study groups (i.e., early vs. middle vs. late responders) and differential trajectories along the OGTT (i.e., 0 vs. 60 vs. 120 min). Preliminary data processing included the removal of variables containing more than 20% missing values, kNN imputation of remaining missing data, removal of non-informative variables based on the interquartile range, logarithmic transformation, and Pareto scaling. Afterward, orthogonal partial least squares discriminant analysis (OPLS-DA) was applied to explore the discriminant power of metabolomics data in a multivariate manner (i.e., Variable Importance for the Projection parameter greater than 1). To evaluate the significance of the associations observed in multivariate models, we then employed analysis of variance (ANOVA) with Fisher LSD post hoc tests. Complementarily, additional linear models with covariate adjustment were computed to control for the influence of BMI as a potential confounding factor. All the statistical analyses were conducted using the MetaboAnalyst 5.0 web tool (https://www.metaboanalyst.ca/).

## Results

The study population consisted of 76 children with obesity and concomitant IR, whose demographic, anthropometric, and biochemical characteristics are summarized in Table 1. The three study groups had similar age, sex, and BMI distributions. As defined by stratification criteria, different post-challenge insulin levels were observed depending on the secretion pattern, with early, middle, and late peak groups being characterized by higher insulin levels at 30, 60, and 90 min along the OGTT, respectively. Children with delayed insulinemia also showed higher blood pressure, HOMA-IR scores, and glucose levels at the end of the OGTT curve (i.e., 90 and 120 min). Although not reaching statistical significance, subjects with a late insulin peak tended to have lower KIDMED scores (*p* = 0.15).

**Table 1:** Demographic, anthropometric, and biochemical characteristics of the study population

	Early peak group	Middle peak group	Late peak group	*p* value
N	18	19	39	
Sex (% male)	66.7	62.1	61.5	NS
Age (years)	10.9 ± 2.4	12.0 ± 1.6	11.7 ± 2.0	NS
Weight (kg)	70.8 ± 21.7	71.3 ± 19.2	72.0 ± 16.3	NS
Weight (Z-score)	5.0 ± 1.5	4.6 ± 1.1	4.7 ± 2.1	NS
Body mass index (kg/m^2^)	31.0 ± 5.9	28.5 ± 4.9	30.5 ± 5.3	NS
Body mass index (Z-score)	4.5 ± 1.9	3.7 ± 1.3	4.2 ± 2.0	NS
Systolic blood pressure (mmHg)	115.3 ± 10.9^a^	117.3 ± 11.8^a^	124.2 ± 10.8^b^	5.4 × 10^−3^
Diastolic blood pressure (mmHg)	74.5 ± 6.6^a^	74.1 ± 6.7^a^	77.9 ± 6.4^b^	3.7 × 10^−2^
Glucose, t = 0 min (mg/dL)	86.1 ± 9.4	85.3 ± 7.9	84.4 ± 9.2	NS
Glucose, t = 30 min (mg/dL)	148.8 ± 22.1	141.3 ± 28.6	139.9 ± 25.3	NS
Glucose, t = 60 min (mg/dL)	123.9 ± 17.8	134.5 ± 24.4	141.7 ± 33.0	NS
Glucose, t = 90 min (mg/dL)	116.5 ± 11.5^a^	108.6 ± 23.7^a^	139.0 ± 31.0^b^	2.2 × 10^−5^
Glucose, t = 120 min (mg/dL)	114.1 ± 13.1^a^	113.8 ± 23.9^a^	136.4 ± 27.8^b^	1.4 × 10^−3^
Insulin, t = 0 min (µU/mL)	20.5 ± 5.3	19.9 ± 5.9	23.3 ± 12.4	NS
Insulin, t = 30 min (µU/mL)	223.4 ± 117.0^a^	122.4 ± 51.9^b^	130.7 ± 65.5^b^	5.3 × 10^−4^
Insulin, t = 60 min (µU/mL)	154.8 ± 92.1^a^	196.8 ± 68.3^b^	152.5 ± 80.7^a^	6.0 × 10^−3^
Insulin, t = 90 min (µU/mL)	143.6 ± 102.2^a^	117.5 ± 59.4^a^	196.1 ± 134.2^b^	7.9 × 10^−3^
Insulin, t = 120 min (µU/mL)	143.7 ± 93.0	136.5 ± 87.0	191.6 ± 103.1	NS
HOMA-IR score	4.3 ± 1.4^a^	4.2 ± 1.3^a^	4.9 ± 3.0 ^b^	4.9 × 10^−2^
KIDMED score	7.5 ± 1.4	6.2 ± 2.5	6.0 ± 2.2	NS

Results are expressed as mean ± standard deviation (except for sex, expressed as percentage). Superscript letters within each row indicate significant differences between groups that are marked with different letters, according to the post hoc Fisher LSD test (*p* < 0.05). NS, non-significant

After multivariate modeling (Figure S1), a total number of 20 plasmatic and 12 erythroid differential metabolites were identified between the three study groups at fasting, as detailed in Tables S1-S2. The statistical significance of most of these associations was maintained after adjusting for BMI as a confounding factor. Compared to early responders, many of these differential metabolites showed higher levels in both plasma and erythrocytes of children with late and, to a lesser extent, middle insulin peaks, including energy-related metabolites (e.g., Krebs intermediates, acyl-carnitines, free fatty acids), amino acids, markers of oxidative stress, bile acids, steroid hormones, and phospholipids. In contrast, a lower content was detected in plasma cortisol and 3-(4-hydroxyphenyl)propionic acid, as well as in erythroid 3-hydroxy-trimethyllysine, aspartic acid, hypoxanthine, and 4-oxononenal glutathione. Notably, children with late and middle insulin peaks had similar levels for most of these metabolites. However, some metabolic changes were found to be exacerbated among children in the late peak group (e.g., alanine, cortisol), which could be regarded as sensitive markers of IR severity.

Complementary analysis of samples collected along the OGTT demonstrated a profound impact of this challenge test on individuals’ metabolism, at both systemic and erythroid levels (Tables S3-S4, Figures S2-S3). Acute carbohydrate overload primarily provoked an increase in glycolytic intermediates (glucose and derivatives, lactic acid) and hippuric acid in the three study groups. This was in turn accompanied by lower levels of other metabolites related to mitochondrial energy homeostasis, such as ketone bodies, acyl-carnitines, hydroxylated fatty acids, free fatty acids, and amino acids. The OGTT also induced a decrease of corticosteroids in plasma and cysteinyl-glycine in erythrocytes, whereas circulating L-carnitine tended to increase. Moreover, it should be noted that distinct postprandial trajectories were observed between the study groups. Delayed insulin secretion in response to the OGTT was associated with a more pronounced increase in glycolytic metabolites, as well as in blunted decline of some fatty acids and amino acids. In contrast, the OGTT-induced decrease in erythroid cysteinyl-glycine was exclusively detected among children with middle and late insulin peaks.

## Discussion

**Fig. 1:**
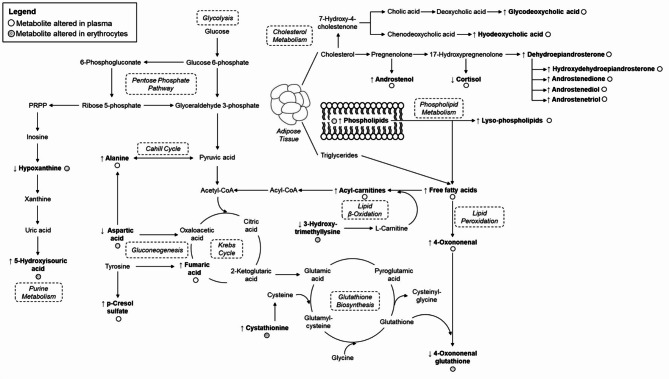
Overview of differential metabolites detected in plasma and erythrocytes

Although it is recognized that IR is one of the most prevalent and pathogenically relevant metabolic complications underlying childhood obesity and related comorbidities, there is international consensus on the need to delve deeper into its pathophysiology to identify better biomarkers for diagnosis and classification [3]. In this respect, recent studies suggest that the shape of insulin curves along an OGTT could be a surrogate indicator of IR severity, which would facilitate stratifying populations a priori considered to be homogeneous into distinct phenotypic subtypes. Concurring with existing evidence [6], we found children with delayed OGTT-induced insulinemia to exhibit aggravated cardiometabolic derangements, as reflected in higher blood pressure, glycemia and HOMA-IR scores when compared to early responders (Table 1). Based on this rationale, fasting plasma and erythrocyte samples were subjected to metabolomics analysis to identify metabolite alterations associated with disease severity, as schematized in Fig. 1. Despite our three study groups were matched on BMI, as some studies have reported slight weight increments in the late insulin peak group [6], additional statistical models adjusted for BMI were computed to discard the potential influence of this confounding variable and, thus, to identify metabolites robustly associated with the distinct IR metabotypes.

Obesity and IR have primarily been associated with a shift toward hypoxic metabolism of carbohydrates to counteract dysfunctions in mitochondrial pathways [19]. Herein, we interestingly found that energy-related disturbances were exacerbated among children with late and, to a lesser extent, middle insulin peaks, thereby reinforcing the hypothesis that the pattern of insulin secretion during an OGTT might serve as a reliable predictor of individuals’ metabolic health. Higher circulating levels of fumaric acid could indicate impairments in the Krebs cycle, whereas decreased erythroid 3-hydroxy-trimethyllysine (a precursor in the biosynthesis of L-carnitine) and increased plasma stearoyl-L-carnitine suggest disrupted β-oxidation of lipids, in line with other studies [19]. In this vein, elevated plasma free fatty acids have been described to be the result of various interrelated mechanisms, including incomplete clearance through β-oxidation, release from adipose tissue, and higher dietary intake [20]. These lipids may in turn stimulate IR by worsening β-cell function and inhibiting sensitivity of target cells, which could account for the higher levels detected among subjects with delayed insulin response. Along with these changes reflecting mitochondrial deficits, increments in blood alanine further support profound failures in energy homeostasis, as this amino acid may serve as a precursor of pyruvic acid through the Cahill cycle and, consequently, as a marker of boosted anaerobic catabolism [19]. This was accompanied by reduced erythroid content of aspartic acid, which participates in the synthesis of alanine and oxaloacetic acid, thereby establishing a plausible link between IR-related disturbances in energy and amino acid metabolisms [21]. This inefficient utilization of nutrients is well-known to be one of the most important triggers of oxidative stress, as excess supply of substrates into oxidative catabolic pathways may overload the mitochondrial electron transport chain and cause sustained generation of free radicals [22]. Thus, in line with the aforementioned worsening in energy metabolism, children with late hyperinsulinemia also showed more pronounced changes in several oxidative stress markers at both systemic and cellular levels. Increased lipid peroxidation [23] and defective neutralizing capacity of the glutathione system [24] have been proven as typical hallmarks of oxidative stress in obesity and IR, which could explain our results on higher levels of 4-oxononenal (in plasma) and simultaneous reduction of its detoxification product, 4-oxononenal glutathione (in erythrocytes). Similarly, this exacerbated stress was reflected in accelerated purine degradation, with increased erythroid production of 5-hydroxyisouric acid from its precursor hypoxanthine [25]. Under this stressful milieu, proinflammatory microbiota is known to modulate oxidative stress and chronic inflammation by releasing uremic toxins, such as p-cresol sulfate, which may contribute to the development of metabolic syndrome [26].

Complementarily, our results suggest a close relationship between the insulin secretion pattern and various lipids classes, including cholesterol derivatives and phospholipids. Obesity stimulates cholesterol metabolism toward the production of bile acids to promote fat absorption [27], as well as the production of sex hormones through a complex meshwork of interrelated mechanisms, such as secretion of gonadotropin-releasing hormone, over-expression of adrenocorticotropic hormone, and reduction of sex hormone-binding globulin levels [28]. This release of steroid compounds may in turn bidirectionally modulate lipid homeostasis, inflammation, and insulin function, thereby creating a vicious pathological cycle [29]. Accordingly, these lipid perturbations would be expected to worsen among subjects with severe IR, as we report here. In contrast, plasma cortisol levels were lower in late responders when compared to children with early and middle insulin peaks. This apparently contradictory findings could be attributed to dysregulations in the circadian rhythm, in line with previous studies describing a flattened cortisol profile in subjects with diabetes, as mirrored in lower morning and higher evening levels of this corticosteroid [30]. Regarding phospholipid species, delayed insulinemia was associated with higher plasma content of lyso-phospholipids and increased erythroid levels of phosphatidylcholines and phosphatidylethanolamines. Although the existing literature on the association between phospholipids, obesity, and IR is controversial, some authors have reported a similar overproduction of circulating lyso-phospholipids due to enhanced phospholipase activity [31]. On the other hand, the raise in erythroid phospholipids could be allocated to various intertwined mechanisms, in line with previous data demonstrating a positive association between fasting insulin, HOMA-IR scores, and phospholipids in erythrocyte membranes [32]. In that study, the authors hypothesized that IR could affect phospholipid homeostasis in different ways, such as by altering their dynamic exchange between cell membranes and plasma lipoproteins, regulating the production of molecules that stimulate cell import (e.g., chemokine connective tissue-activating peptide III), and enhancing biosynthetic pathways. Altogether, lipid metabolism could represent a cornerstone in the metabolic derangements occurring in IR and childhood obesity, as presumable considering that adipocytes are physiologically insulin resistant cells.

Finally, metabolomics analysis of fasting samples also evidenced that subjects showing a late insulin peak had higher plasma levels of 1-methylhistidine together with decreased 3-(4-hydroxyphenyl)propionic acid, which are reliable markers reflecting the intake of meat and plant-based foods, respectively [33]. Furthermore, we detected an increased erythroid content of cystathionine, a metabolite that has traditionally been linked to vitamin B6 deficiency in obesity [34] and diabetes [35]. As these findings suggest the involvement of nutritional factors in IR pathophysiology, the KIDMED questionnaire was employed to evaluate diet quality of the study population. Interestingly, we found that children with delayed insulinemia tended to have lower KIDMED scores (not reaching statistical significance), indicative of lesser preference for Mediterranean diet items (e.g., fruits, vegetables) and greater adherence to Western diets (e.g., red meat), which could explain our metabolomics results. This concurs with a recent study describing that children with impaired response against an OGTT had lower blood arsenic levels and self-reported lower intake of seafood products, which is another pivotal component of the Mediterranean diet [8]. Therefore, unhealthy dietary habits stand out as an important risk factor presumably modulating the onset and severity of IR in children with obesity.

**Fig. 2:**
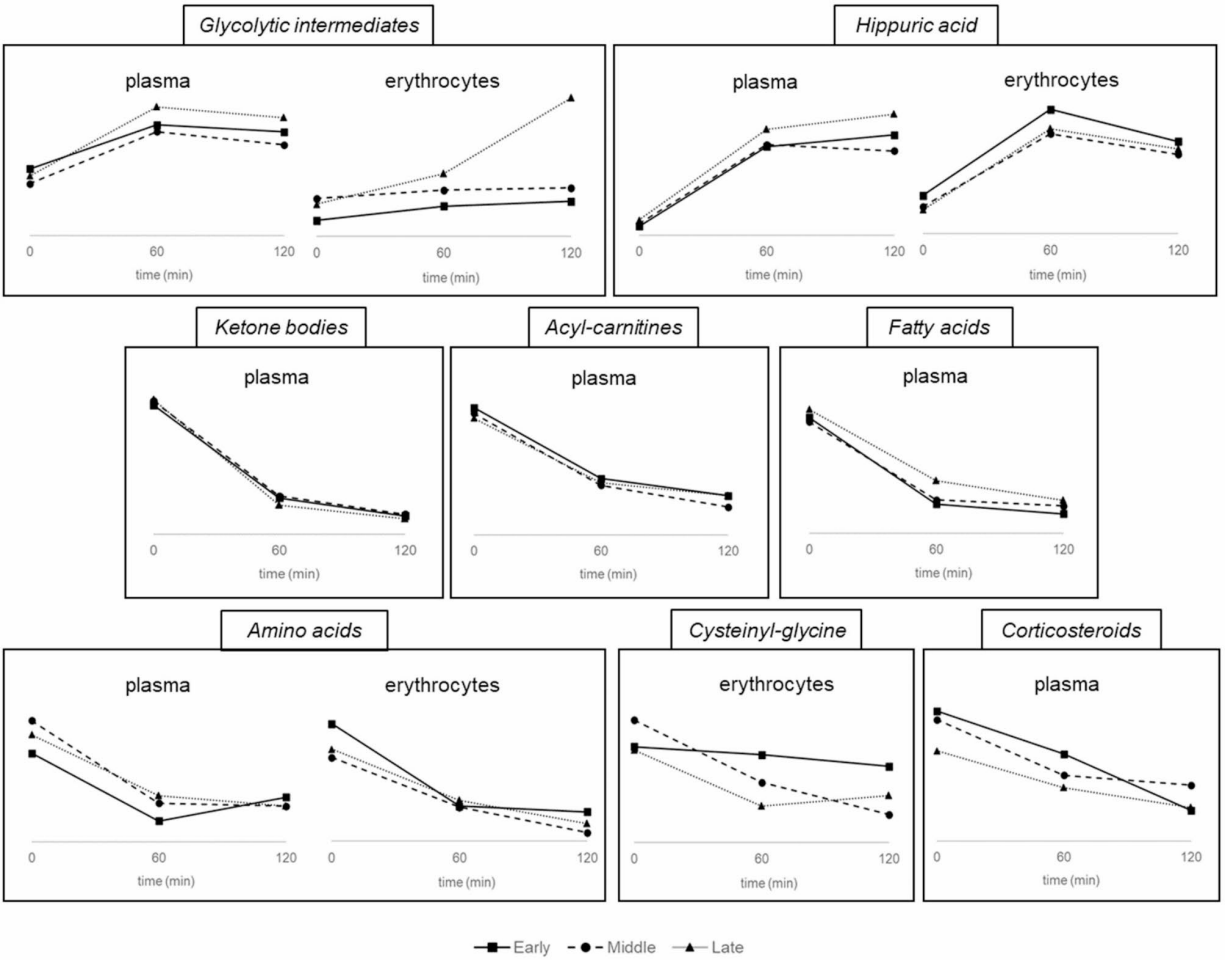
Schematic representation of time-dependent trajectories showing the effect of the oral glucose tolerance test in metabolite levels

After identifying distinctive metabotypes of IR, as assessed by inspecting the morphology of insulin curves, our second major aim was to decipher the influence of disease severity degree in individuals’ metabolic flexibility in response to a challenge test. As expected, the OGTT caused a transient increase in plasma and erythroid levels of various glycolytic intermediates (i.e., glucose and derivatives, lactic acid) and hippuric acid, this latter resulting from the metabolization of the preservative benzoate contained in glucose syrups [36]. This was accompanied by downregulated levels of ketone bodies, acyl-carnitines, hydroxylated fatty acids, free fatty acids, and amino acids, suggesting the inhibition of mitochondrial energy pathways (i.e., ketogenesis, β-oxidation, proteolysis, and gluconeogenesis) with the aim to manage the glucose overload via glycolysis [36, 37]. More interestingly, the comparison of the three study groups enabled us discovering specific trajectories in these metabolic adaptations, as schematized in Fig. 2. Children with delayed OGTT-induced insulinemia showed a more pronounced increase of glycolytic products, together with blunted decline in some fatty acids and amino acids in plasma and erythrocytes. These findings are in accordance with other studies demonstrating that IR may disrupt metabolic flexibility in people with obesity [38, 39], impairments that are expected to be aggravated by disease severity. The challenge test also provoked a postprandial reduction of erythroid cysteinyl-glycine, an intermediate in glutathione biosynthesis, as previously reported by Wopereis et al. [40]. However, it is noteworthy that levels of this peptide were exclusively altered among children with middle and late insulin peaks, which could be indicative of their compromised capacity to fight against oxidative stress. To conclude, plasma levels of cortisol and other corticosteroids were also found to decline along the OGTT, trends that could be allocated to diurnal falls related to the circadian rhythm rather than glucose-induced metabolic adaptations [41].

This study is majorly strengthened by the use, for the first time, of a novel approach to better characterize IR in children with obesity. Unlike most of the existing metabolomics literature, where the role of IR in modulating obesity-related metabolic disturbances has usually been explored by looking for associations with surrogate markers (e.g., HOMA-IR), we have demonstrated that the shape of OGTT-induced insulin curves is a reliable measure to stratify subjects according to disease severity (i.e., early vs. late responders). In this respect, the recruitment of a well-characterized population, matched for relevant covariates such as BMI, enabled us to minimize the potential interference of confounding factors and, thus, to deepen into IR pathophysiology. Another strength of this study was the complementary analysis of fasting and postprandial biological samples, which stands out as a suitable strategy to better understand the impact of IR on individuals’ homeostasis (i.e., metabotypes) and their capacity to properly face external stressors (i.e., metabolic flexibility). To this end, parallel research in plasma and erythrocytes has provided the opportunity to characterize the metabolic alterations occurring in the crosstalk between IR and obesity in a comprehensive manner, at both systemic and cellular levels. On the other hand, it should be stressed that stratification according to the insulin secretion profile inherently limited the statistical power of the comparisons conducted, so further studies in larger and independent cohorts are needed for validation purposes and to define robust biomarkers with clinical applicability. Furthermore, the observational nature of our study design has precluded disentangling whether IR could be a cause or a consequence of the metabolic disorders herein described.

## Conclusions

To our knowledge, this is the first study exploring distinctive metabotypes of IR in children with obesity. In line with recent studies, we found that insulin peak timing along an OGTT is a reliable predictor of individuals’ health, with subjects exhibiting late response being characterized by exacerbated cardiometabolic derangements. When studying fasting plasma and erythrocytes, children with delayed insulin secretion showed more pronounced changes in typical IR-related disturbances in energy homeostasis (e.g., Krebs cycle, lipid β-oxidation), oxidative control (e.g., glutathione synthesis, purine degradation), cholesterol metabolism (e.g., synthesis of bile acids and steroid hormones), and membrane phospholipids. This evidences that fasting metabolite determinations could serve as predictive biomarkers of IR severity, without the need of performing a time-consuming and invasive OGTT. Moreover, integrated metabolomics and dietary assessment suggested the involvement of unhealthy nutritional habits as a potential risk factor in modulating the onset of IR in children with obesity. As a complementary approach, the study of postprandial samples collected along the OGTT evidenced that IR may have a deleterious impact on metabolic flexibility, as children with late insulin peak were unable to properly regulate energy metabolism and antioxidant defenses to face the transient metabolic stress provoked by glucose overload. In conclusion, this study demonstrates the potential of metabotyping to identify distinct phenotypic subtypes in complex disorders such as IR, and opens the door to establish better markers for its diagnosis and classification.

### Electronic supplementary material

Supplementary Material 1

## Data Availability

The datasets used and/or analysed during the current study are available from the corresponding author on reasonable request.

## Abbreviations

BMI: Body mass index
HOMA-IR: Homeostatic model assessment of insulin resistance
IR: Insulin resistance
OGTT: Oral glucose tolerance test
