# Supplementary material for: Identifying metabotypes of insulin resistance severity in children with metabolic syndrome
Source: Cardiovasc Diabetol. 2024 Aug 27;23:315. doi: 10.1186/s12933-024-02412-x (PMC11348533; doi:10.1186/s12933-024-02412-x)
Supplement: Supplementary file 1 — Supplementary Material 1 [file 12933_2024_2412_MOESM1_ESM.docx]

**Identifying metabotypes of insulin resistance severity in children with metabolic syndrome**

Álvaro González-Domínguez ^1,2^, Jesús Domínguez-Riscart ^1,3^, Otto Savolainen ^4^, Alfonso Lechuga-Sancho ^1,3,5^, Rikard Landberg ^4^, Raúl González-Domínguez ^1,*^

^1^ Instituto de Investigación e Innovación Biomédica de Cádiz (INiBICA), Hospital Universitario Puerta del Mar, Universidad de Cádiz. 11009 Cádiz, Spain. ^2^ Division of Liver Diseases, Icahn School of Medicine at Mount Sinai. 10029 New York, USA. ^3^ Unidad de Endocrinología Pediátrica y Diabetes, Servicio de Pediatría, Hospital Universitario Puerta del Mar. 11009 Cádiz, Spain. ^4^ Division of Food and Nutrition Science, Department of Life Sciences, Chalmers University of Technology. SE-412 96 Gothenburg, Sweden. ^5^ Departamento Materno Infantil y Radiología, Facultad de Medicina, Universidad de Cádiz. 11009 Cádiz, Spain.

*Corresponding author: [raul.gonzalez@inibica.es](mailto:raul.gonzalez@inibica.es)


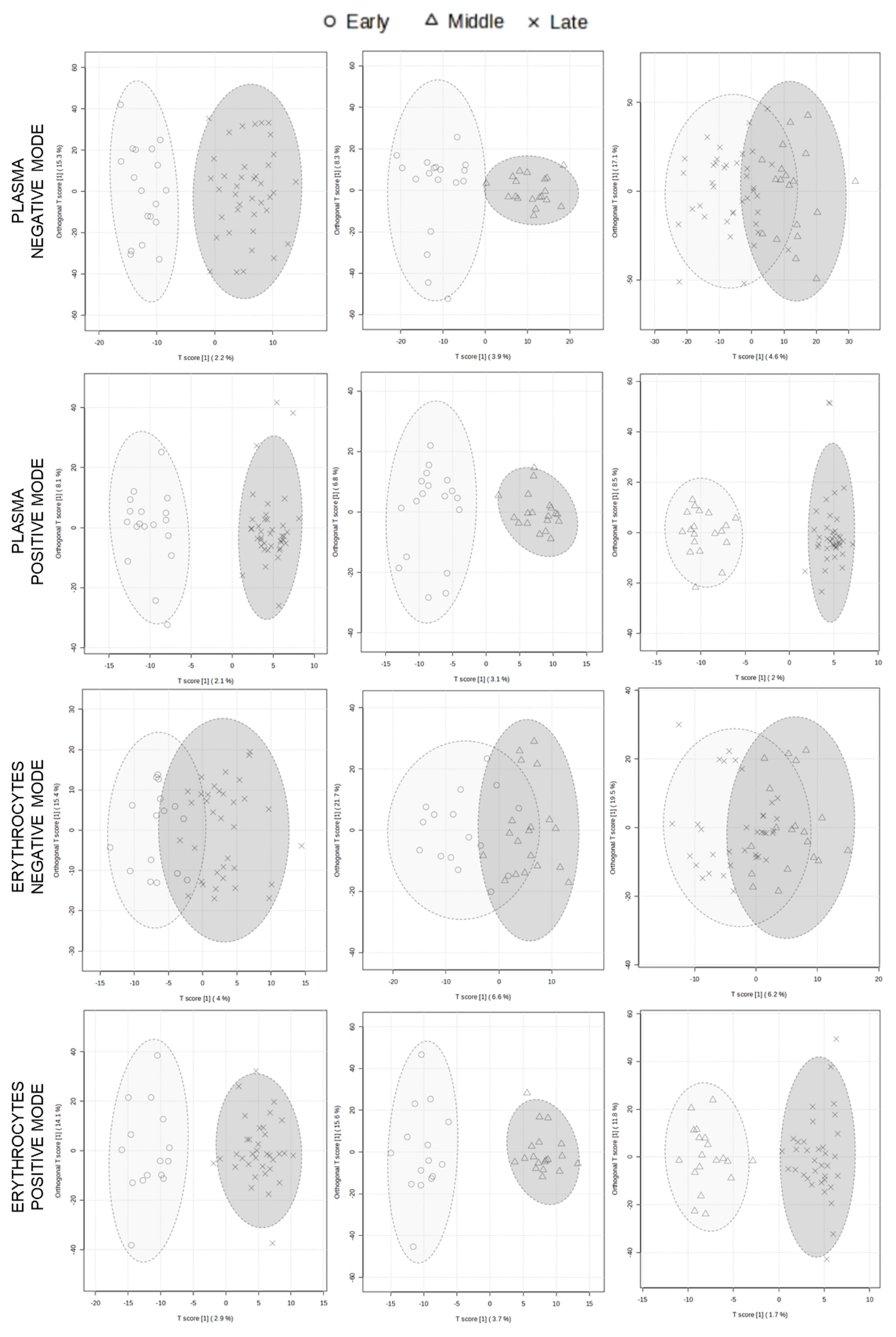


**Figure S1.** Orthogonal partial least squares discriminant analysis (OPLS-DA) scores plots showing the separation between the study groups (i.e., early, middle, and late insulin peak) using plasma and erythroid metabolomics data at fasting.


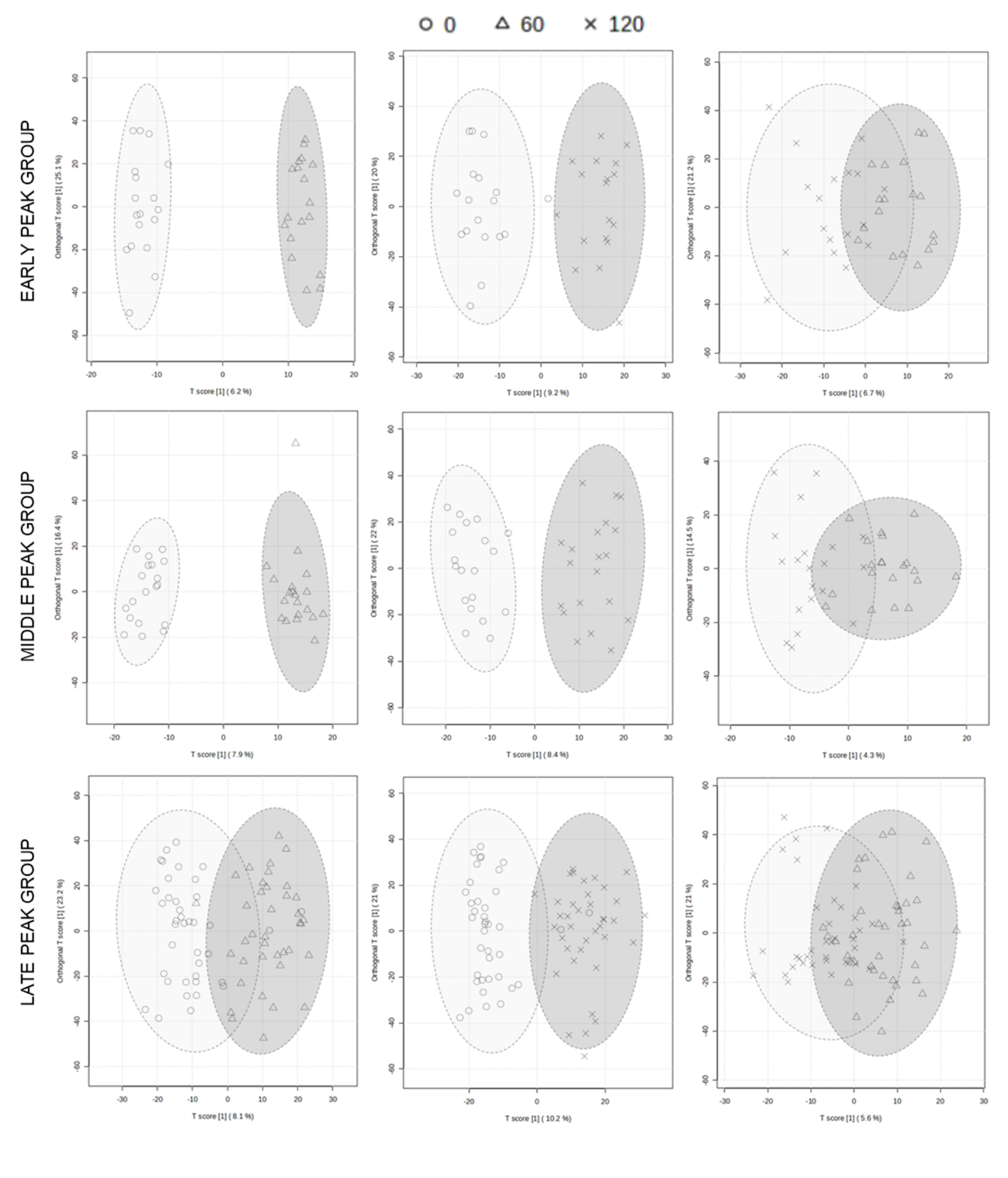


**Figure S2.** Orthogonal partial least squares discriminant analysis (OPLS-DA) scores plots showing the separation between the study groups (i.e., 0 min, 60 min, and 120 min) using plasma metabolomics data at along the oral glucose tolerance test.


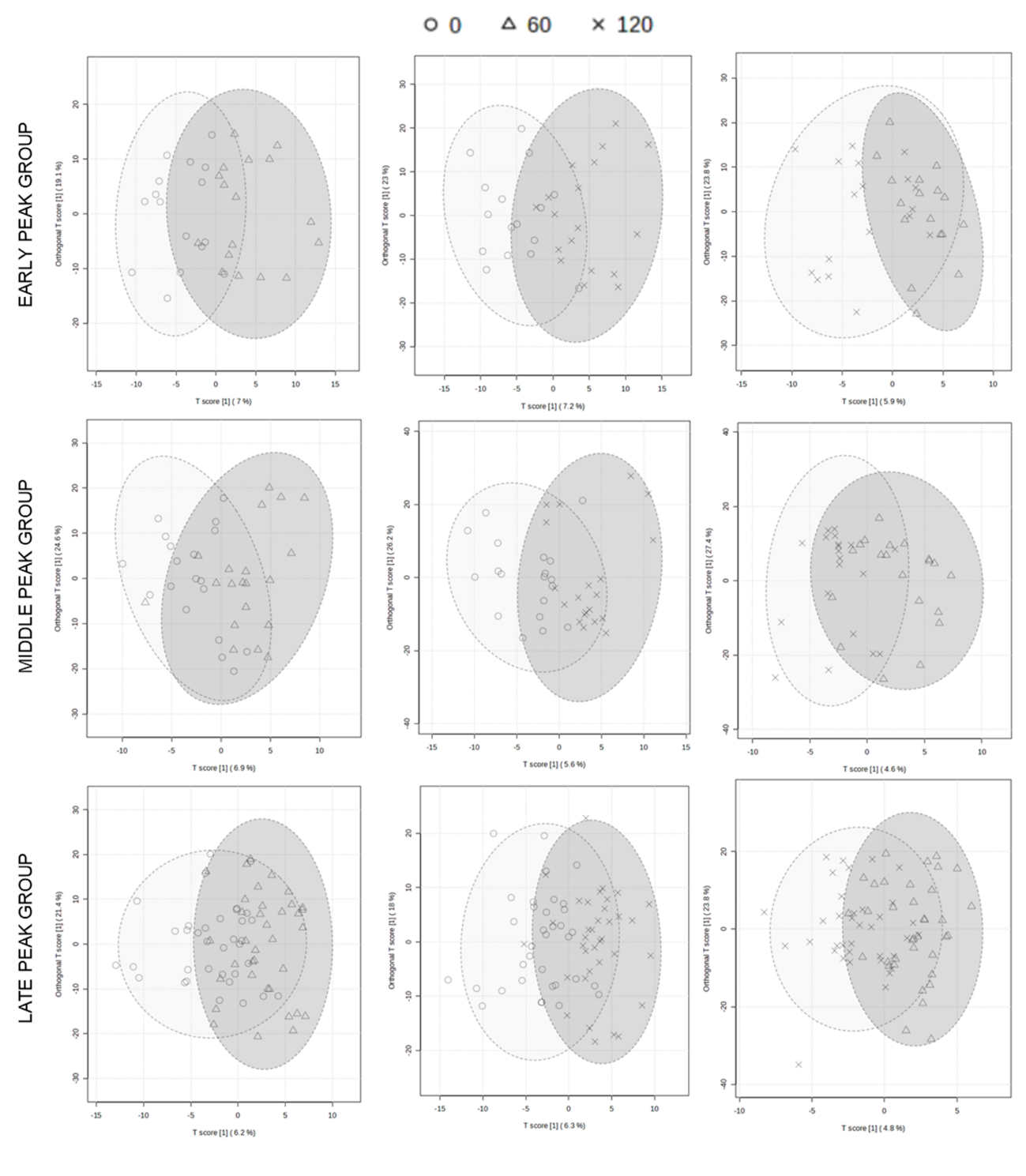


**Figure S3.** Orthogonal partial least squares discriminant analysis (OPLS-DA) scores plots showing the separation between the study groups (i.e., 0 min, 60 min, and 120 min) using erythroid metabolomics data at along the oral glucose tolerance test.

**Table 1.** Differential plasma metabolites between children with late insulin peak, middle insulin peak, and early insulin peak. * Indicates significant differences according to the post-hoc Fisher LSD test (p < 0.05).

| **Metabolite** | **Mass (Da)** | **RT (min)** | **MSI level** | **p value** | **p value (adjusted)** | **Fold changes** | | |
| --- | --- | --- | --- | --- | --- | --- | --- | --- |
|  |  |  |  |  |  | **Late vs. Early** | **Middle vs. Early** | **Late vs. Middle** |
| *Krebs intermediates* | | | | | | | | |
| Fumaric acid | 116.0110 | 0.85 | 1 | 3.4×10^-2^ | 2.8×10^-2^ | 1.77* | 1.81* | 0.98 |
| *Carnitine-related metabolites* | | | | | | | | |
| Stearoyl-L-carnitine | 427.3662 | 6.45 | 1 | 1.9×10^-2^ | 2.1×10^-2^ | 1.09* | 1.13* | 0.96 |
| *Free fatty acids* | | | | | | | | |
| Arachidonic acid | 304.2402 | 7.07 | 1 | 4.9×10^-2^ | 4.9×10^-2^ | 1.12* | 1.06 | 1.05 |
| *Amino acids & derivatives* | | | | | | | | |
| L-Alanine | 89.0477 | 0.73 | 1 | 4.3×10^-2^ | 4.8×10^-2^ | 1.23* | 0.98 | 1.25* |
| p-Cresol sulfate | 188.0143 | 3.13 | 1 | 4.9×10^-2^ | 1.5×10^-1^ | 1.21* | 1.36* | 0.89 |
| *Oxidative stress markers* | | | | | | | | |
| 4-Oxononenal | 154.0994 | 3.93 | 2 | 3.7×10^-2^ | 3.6×10^-2^ | 1.25* | 1.09 | 1.15 |
| *Bile acids* | | | | | | | | |
| Glycodeoxycholic acid sulfate | 529.2709 | 6.02 | 1 | 1.9×10^-2^ | 3.1×10^-2^ | 1.67* | 2.18* | 0.77 |
| Hyodeoxycholic acid | 392.2927 | 6.19 | 1 | 4.2×10^-2^ | 4.3×10^-2^ | 1.77* | 1.94* | 0.91 |
| *Steroid hormones* | | | | | | | | |
| Dehydroepiandrosterone sulfate | 368.1657 | 5.24 | 1 | 1.8×10^-2^ | 1.9×10^-2^ | 1.56* | 1.87* | 0.83 |
| Hydroxydehydroepiandrosterone sulfate | 384.1607 | 4.62 | 3 | 2.4×10^-2^ | 1.8×10^-2^ | 2.17* | 2.15* | 1.01 |
| Androstenedione sulfate | 366.1501 | 4.90 | 3 | 3.9×10^-2^ | 2.7×10^-2^ | 1.38* | 1.54* | 0.89 |
| Androstenediol sulfate | 370.1814 | 5.03 | 3 | 3.3×10^-2^ | 3.5×10^-2^ | 1.41* | 1.48* | 0.95 |
| Androstenetriol / Hydroxyandrosterone sulfate | 386.1763 | 4.73 | 3 | 4.5×10^-2^ | 3.2×10^-2^ | 1.74* | 2.02* | 0.86 |
| Androstenol glucuronide | 450.2646 | 6.66 | 3 | 4.2×10^-3^ | 2.8×10^-2^ | 1.32* | 1.14 | 1.16 |
| Cortisol | 362.2093 | 5.00 | 1 | 2.1×10^-2^ | 2.5×10^-2^ | 0.75* | 0.99 | 0.76* |
| *Phospholipids* | | | | | | | | |
| LPC(16:1) | 493.3168 | 6.67 | 3 | 4.8×10^-2^ | 3.3×10^-2^ | 1.06* | 1.07* | 0.99 |
| LPC(18:2) | 519.3325 | 6.90 | 3 | 1.3×10^-2^ | 1.0×10^-2^ | 1.07* | 1.09* | 0.98 |
| LPC(20:4) | 543.3325 | 6.88 | 3 | 2.5×10^-2^ | 5.9×10^-2^ | 1.14* | 1.27* | 0.90 |
| *Diet-related metabolites* | | | | | | | | |
| 1-Methylhistidine | 169.0851 | 0.65 | 1 | 1.1×10^-2^ | 4.9×10^-2^ | 1.23* | 1.06 | 1.16 |
| 3-(4-Hydroxyphenyl)propionic acid | 166.0630 | 3.70 | 1 | 2.7×10^-2^ | 3.4×10^-2^ | 0.88* | 0.88* | 0.99 |

**Table 2.** Differential erythroid metabolites between children with late insulin peak, middle insulin peak, and early insulin peak. * Indicates significant differences according to the post-hoc Fisher LSD test (p < 0.05).

| **Metabolite** | **Mass (Da)** | **RT (min)** | **MSI level** | **p value** | **p value (adjusted)** | **Fold changes** | | |
| --- | --- | --- | --- | --- | --- | --- | --- | --- |
|  |  |  |  |  |  | **Late vs. Early** | **Middle vs. Early** | **Late vs. Middle** |
| *Carnitine-related metabolites* | | | | | | | | |
| 3-Hydroxy-trimethyllysine | 205.1552 | 0.61 | 2 | 8.6×10^-3^ | 1.0×10^-2^ | 0.84* | 0.90 | 0.93 |
| *Amino acids & derivatives* | | | | | | | | |
| L-Aspartic acid | 133.0375 | 0.69 | 1 | 1.2×10^-2^ | 1.5×10^-2^ | 0.75* | 0.71* | 1.06 |
| Cystathionine | 222.0674 | 1.25 | 2 | 1.5×10^-2^ | 2.1×10^-2^ | 1.33* | 1.30* | 1.02 |
| *Oxidative stress markers* | | | | | | | | |
| 5-Hydroxyisouric acid | 184.0232 | 0.94 | 2 | 4.6×10^-2^ | 3.8×10^-2^ | 1.34* | 1.17 | 1.14 |
| Hypoxanthine | 136.0385 | 1.23 | 1 | 6.8×10^-3^ | 2.3×10^-2^ | 0.77* | 0.66* | 1.17 |
| 4-Oxononenal glutathione | 461.1832 | 4.27 | 2 | 3.8×10^-2^ | 1.2×10^-2^ | 0.15* | 0.16* | 0.94 |
| *Phospholipids* | | | | | | | | |
| PC(16:0/20:4) | 781.5622 | 8.47 | 3 | 4.9×10^-2^ | 5.9×10^-2^ | 1.07* | 1.11* | 0.96 |
| PE(18:1/20:4) | 765.5309 | 8.57 | 3 | 5.3×10^-3^ | 9.8×10^-3^ | 1.06* | 1.09* | 0.97 |
| PE(P-18:0/22:6) | 775.5516 | 8.90 | 3 | 3.1×10^-2^ | 3.1×10^-2^ | 1.03* | 1.08 | 0.96 |
| PE(P-18:1/22:4) | 777.5672 | 9.27 | 3 | 1.3×10^-2^ | 1.8×10^-2^ | 1.17* | 1.28* | 0.92 |
| PE(P-18:1/18:1) | 727.5516 | 9.35 | 3 | 2.5×10^-3^ | 1.5×10^-3^ | 1.36* | 1.41* | 0.97 |
| PE(P-18:0/20:4) | 751.5516 | 9.35 | 3 | 1.8×10^-3^ | 9.1×10^-4^ | 1.19* | 1.27* | 0.94 |

**Table 3.** Differential plasma metabolites along the oral glucose tolerance test in children with late insulin peak, middle insulin peak, and early insulin peak. * Indicates significant differences according to the post-hoc Fisher LSD test (p < 0.05).

| **Metabolite** | **Mass (Da)** | **RT (min)** | **MSI level** | **group** | **p value** | **Fold changes** | | |
| --- | --- | --- | --- | --- | --- | --- | --- | --- |
|  |  |  |  |  |  | **60 vs. 0** | **120 vs. 0** | **120 vs. 60** |
| *Glycolytic intermediates* | | | | | | | | |
| D-Glucose (or isomer) | 180.0634 | 0.72 | 1 | E | 2.5×10^-3^ | 1.18* | 1.18* | 1.00 |
|  |  |  |  | M | 3.2×10^-2^ | 1.25* | 1.11 | 0.88 |
|  |  |  |  | L | 4.0×10^-11^ | 1.33* | 1.25* | 0.94 |
| D-Glucose 6-sulfate (or isomer) | 260.0202 | 0.71 | 3 | E | 3.9×10^-2^ | 1.30* | 1.25* | 0.96 |
|  |  |  |  | M | 1.1×10^-4^ | 1.39* | 1.29* | 0.93 |
|  |  |  |  | L | 1.3×10^-11^ | 1.49* | 1.41* | 0.95 |
| Lactic acid | 90.0317 | 0.89 | 1 | E | NS | 1.20 | 1.11 | 0.93 |
|  |  |  |  | M | 4.1×10^-2^ | 1.42* | 1.16 | 0.81 |
|  |  |  |  | L | 9.6×10^-5^ | 1.45* | 1.19 | 0.83 |
| *Ketone bodies* | | | | | | | | |
| 3-Hydroxybutyric acid | 104.0473 | 1.46 | 1 | E | 1.8×10^-2^ | 0.37* | 0.25* | 0.67 |
|  |  |  |  | M | 3.5×10^-5^ | 0.37* | 0.25* | 0.67 |
|  |  |  |  | L | 5.3×10^-6^ | 0.31* | 0.22* | 0.71 |
| *Carnitine-related metabolites* | | | | | | | | |
| L-Carnitine | 161.1052 | 0.67 | 1 | E | NS | 1.05 | 1.11 | 1.06 |
|  |  |  |  | M | 1.9×10^-2^ | 1.09* | 1.10* | 1.01 |
|  |  |  |  | L | 4.4×10^-7^ | 1.18* | 1.20* | 1.01 |
| Hydroxytetradecadienoyl-L-carnitine | 383.2672 | 5.38 | 3 | E | 4.3×10^-2^ | 0.79* | 0.79* | 1.00 |
|  |  |  |  | M | 3.8×10^-2^ | 0.79* | 0.72* | 0.92 |
|  |  |  |  | L | 2.4×10^-4^ | 0.80* | 0.77* | 0.95 |
| Oleoyl-L-carnitine | 425.3505 | 6.32 | 1 | E | 4.4×10^-3^ | 0.88 | 0.75* | 0.85* |
|  |  |  |  | M | 1.3×10^-3^ | 0.89 | 0.75* | 0.85* |
|  |  |  |  | L | 6.5×10^-5^ | 0.85 | 0.78* | 0.92 |
| *Hydroxylated fatty acids* | | | | | | | | |
| Hydroxyoctanoic acid isomer | 160.1099 | 4.72 | 3 | E | 4.5×10^-5^ | 0.59* | 0.56* | 0.96 |
|  |  |  |  | M | 8.2×10^-6^ | 0.62* | 0.54* | 0.86 |
|  |  |  |  | L | 5.1×10^-7^ | 0.64* | 0.55* | 0.87 |
| Hydroxydecanoic acid (isomer 1) | 188.1412 | 4.64 | 3 | E | 1.9×10^-5^ | 0.52* | 0.41* | 0.78 |
|  |  |  |  | M | 4.0×10^-5^ | 0.50* | 0.54* | 1.08 |
|  |  |  |  | L | 7.2×10^-8^ | 0.51* | 0.48* | 0.94 |
| Hydroxydecanoic acid (isomer 2) | 188.1412 | 5.60 | 3 | E | 7.4×10^-10^ | 0.43* | 0.34* | 0.79* |
|  |  |  |  | M | 5.3×10^-10^ | 0.40* | 0.38* | 0.94 |
|  |  |  |  | L | 3.2×10^-18^ | 0.43* | 0.35* | 0.81* |
| Hydroxydecanoic acid (isomer 3) | 188.1412 | 5.81 | 3 | E | 4.4×10^-3^ | 0.73* | 0.64* | 0.88 |
|  |  |  |  | M | 8.9×10^-4^ | 0.69* | 0.62* | 0.89 |
|  |  |  |  | L | 1.4×10^-6^ | 0.74* | 0.65* | 0.88 |
| Hydroxydecenoic acid | 186.1256 | 5.54 | 3 | E | 5.9×10^-3^ | 0.57* | 0.48* | 0.85 |
|  |  |  |  | M | 7.0×10^-3^ | 0.67* | 0.50* | 0.75 |
|  |  |  |  | L | 6.0×10^-5^ | 0.80* | 0.56* | 0.70* |
| 3-Hydroxylauric acid | 216.1725 | 6.16 | 1 | E | 3.8×10^-10^ | 0.42* | 0.36* | 0.86 |
|  |  |  |  | M | 1.8×10^-9^ | 0.42* | 0.42* | 1.00 |
|  |  |  |  | L | 7.5×10^-22^ | 0.45* | 0.37* | 0.81* |
| Hydroxydodecenoic acid | 214.1569 | 5.94 | 3 | E | 8.0×10^-12^ | 0.43* | 0.36* | 0.82 |
|  |  |  |  | M | 1.8×10^-10^ | 0.41* | 0.39* | 0.95 |
|  |  |  |  | L | 2.1×10^-13^ | 0.44* | 0.35* | 0.80* |
| 3-Hydroxymyristic acid | 244.2038 | 6.55 | 1 | E | 3.1×10^-10^ | 0.43* | 0.36* | 0.85 |
|  |  |  |  | M | 1.8×10^-9^ | 0.43* | 0.40* | 0.92 |
|  |  |  |  | L | 5.3×10^-22^ | 0.41* | 0.35* | 0.85* |
| Hydroxytetradecenoic acid | 242.1882 | 6.41 | 3 | E | 1.6×10^-10^ | 0.34* | 0.26* | 0.76* |
|  |  |  |  | M | 3.0×10^-10^ | 0.34* | 0.31* | 0.91 |
|  |  |  |  | L | 3.2×10^-26^ | 0.35* | 0.26* | 0.76* |
| Hydroxytetradecadienoic acid | 240.1725 | 6.20 | 3 | E | 2.4×10^-10^ | 0.34* | 0.25* | 0.74* |
|  |  |  |  | M | 1.1×10^-7^ | 0.33* | 0.30* | 0.90 |
|  |  |  |  | L | 2.0×10^-20^ | 0.36* | 0.25* | 0.71* |
| 16-Hydroxypalmitic acid | 272.2351 | 6.47 | 1 | E | 8.1×10^-10^ | 0.59* | 0.50* | 0.85 |
|  |  |  |  | M | 4.7×10^-10^ | 0.54* | 0.57* | 1.05 |
|  |  |  |  | L | 1.3×10^-23^ | 0.58* | 0.52* | 0.90 |
| 2-Hydroxypalmitic acid | 272.2351 | 6.94 | 1 | E | 1.4×10^-6^ | 0.80* | 0.65* | 0.81* |
|  |  |  |  | M | 2.6×10^-3^ | 0.80* | 0.72* | 0.89 |
|  |  |  |  | L | 2.4×10^-12^ | 0.77* | 0.67* | 0.87* |
| Hydroxyhexadecadienoic acid | 268.2038 | 6.50 | 3 | E | 1.4×10^-7^ | 0.33* | 0.39* | 1.18 |
|  |  |  |  | M | 1.1×10^-6^ | 0.44* | 0.39* | 0.59 |
|  |  |  |  | L | 2.9×10^-13^ | 0.48* | 0.41* | 0.86* |
| 12-Hydroxystearic acid | 300.2664 | 6.75 | 1 | E | 5.8×10^-11^ | 0.57* | 0.46* | 0.80* |
|  |  |  |  | M | 1.1×10^-6^ | 0.55* | 0.59* | 1.07 |
|  |  |  |  | L | 3.3×10^-15^ | 0.56* | 0.48* | 0.87 |
| Hydroxystearic acid isomer | 300.2664 | 7.07 | 3 | E | 2.5×10^-6^ | 0.76* | 0.64* | 0.84* |
|  |  |  |  | M | 5.4×10^-4^ | 0.73* | 0.73* | 1.00 |
|  |  |  |  | L | 1.3×10^-9^ | 0.77* | 0.66* | 0.86* |
| 12-Hydroxyoleic acid | 298.2508 | 6.61 | 1 | E | 1.9×10^-9^ | 0.46* | 0.33* | 0.71* |
|  |  |  |  | M | 1.1×10^-7^ | 0.39* | 0.44* | 1.13 |
|  |  |  |  | L | 1.2×10^-12^ | 0.45* | 0.46* | 1.03 |
| Hydroxyoleic acid isomer | 298.2508 | 6.91 | 3 | E | 3.4×10^-9^ | 0.41* | 0.29* | 0.71* |
|  |  |  |  | M | 1.6×10^-6^ | 0.39* | 0.41* | 1.05 |
|  |  |  |  | L | 3.5×10^-20^ | 0.36* | 0.33* | 0.92 |
| Dihydroxyoctadecenoic acid (DiHOME) | 314.2457 | 6.21 | 3 | E | 5.0×10^-6^ | 0.53* | 0.39* | 0.73 |
|  |  |  |  | M | 5.1×10^-4^ | 0.55* | 0.57* | 1.03 |
|  |  |  |  | L | 1.4×10^-7^ | 0.68* | 0.50* | 0.74* |
| Hydroxyoctadecadienoic acid (HODE) | 296.2351 | 6.76 | 3 | E | 9.2×10^-9^ | 0.36* | 0.33* | 0.92 |
|  |  |  |  | M | 8.6×10^-6^ | 0.41* | 0.44* | 1.05 |
|  |  |  |  | L | 3.0×10^-18^ | 0.37* | 0.28* | 0.75* |
| Hydroxyoctadecatrienoic acid (HOTrE) | 294.2195 | 6.66 | 3 | E | 3.2×10^-8^ | 0.46* | 0.48* | 1.04 |
|  |  |  |  | M | 6.7×10^-5^ | 0.40* | 0.53* | 1.34 |
|  |  |  |  | L | 2.0×10^-15^ | 0.48* | 0.39* | 0.82* |
| *Free fatty acids* | | | | | | | | |
| (5Z,8Z,11Z,14Z,17Z)-Eicosapentaenoic acid | 302.2246 | 6.95 | 1 | E | 3.8×10^-4^ | 0.47* | 0.36* | 0.77 |
|  |  |  |  | M | 1.3×10^-3^ | 0.41* | 0.51* | 1.25 |
|  |  |  |  | L | 3.0×10^-10^ | 0.47* | 0.37* | 0.78 |
| Myristic acid | 228.2089 | 6.95 | 1 | E | 1.9×10^-6^ | 0.42* | 0.45* | 1.09 |
|  |  |  |  | M | 1.9×10^-3^ | 0.59* | 0.60* | 1.02 |
|  |  |  |  | L | 1.2×10^-8^ | 0.58* | 0.59* | 1.03 |
| Linolenic acid | 278.2246 | 6.97 | 1 | E | 9.6×10^-11^ | 0.34* | 0.23* | 0.68* |
|  |  |  |  | M | 2.1×10^-6^ | 0.32* | 0.32* | 1.01 |
|  |  |  |  | L | 4.6×10^-22^ | 0.34* | 0.21* | 0.60* |
| Palmitoleic acid | 254.2246 | 7.02 | 1 | E | 2.1×10^-10^ | 0.28* | 0.20* | 0.71 |
|  |  |  |  | M | 1.1×10^-6^ | 0.33* | 0.27* | 0.84 |
|  |  |  |  | L | 3.3×10^-12^ | 0.44* | 0.29* | 0.66* |
| (4Z,7Z,10Z,13Z,16Z,19Z)-Docosahexaenoic acid | 328.2402 | 7.05 | 1 | E | 3.2×10^-5^ | 0.57* | 0.37* | 0.66* |
|  |  |  |  | M | 5.1×10^-4^ | 0.58* | 0.45* | 0.78 |
|  |  |  |  | L | 1.2×10^-10^ | 0.50* | 0.38* | 0.75* |
| Arachidonic acid | 304.2402 | 7.08 | 1 | E | 7.6×10^-6^ | 0.50* | 0.47* | 0.94 |
|  |  |  |  | M | 9.4×10^-5^ | 0.51* | 0.49* | 0.97 |
|  |  |  |  | L | 2.1×10^-14^ | 0.49* | 0.46* | 0.95 |
| Linoleic acid | 280.2402 | 7.10 | 1 | E | 1.6×10^-11^ | 0.39* | 0.26* | 0.67* |
|  |  |  |  | M | 1.9×10^-6^ | 0.38* | 0.39* | 1.02 |
|  |  |  |  | L | 2.9×10^-21^ | 0.40* | 0.28* | 0.69* |
| (4Z,7Z,10Z,13Z,16Z)-Docosapentaenoic acid | 330.2559 | 7.18 | 1 | E | 2.1×10^-7^ | 0.49* | 0.33* | 0.68* |
|  |  |  |  | M | 6.8×10^-4^ | 0.47* | 0.42* | 0.89 |
|  |  |  |  | L | 8.8×10^-17^ | 0.45* | 0.34* | 0.74* |
| (8Z,11Z,14Z)-Eicosatrienoic acid | 306.2559 | 7.20 | 1 | E | 6.9×10^-9^ | 0.45* | 0.34* | 0.76 |
|  |  |  |  | M | 3.3×10^-4^ | 0.48* | 0.45* | 0.94 |
|  |  |  |  | L | 4.0×10^-19^ | 0.46* | 0.35* | 0.77* |
| Palmitic acid | 256.2402 | 7.22 | 1 | E | 4.0×10^-10^ | 0.43* | 0.43* | 1.01 |
|  |  |  |  | M | 3.5×10^-8^ | 0.39* | 0.41* | 1.04 |
|  |  |  |  | L | 9.5×10^-16^ | 0.47* | 0.45* | 0.95 |
| Oleic acid | 282.2559 | 7.28 | 1 | E | 2.4×10^-12^ | 0.33* | 0.19* | 0.59* |
|  |  |  |  | M | 5.9×10^-7^ | 0.31* | 0.22* | 0.71 |
|  |  |  |  | L | 1.1×10^-22^ | 0.33* | 0.20* | 0.61* |
| Docosatetraenoic acid | 332.2715 | 7.29 | 3 | E | 8.4×10^-9^ | 0.44* | 0.30* | 0.69* |
|  |  |  |  | M | 6.9×10^-5^ | 0.41* | 0.35* | 0.85 |
|  |  |  |  | L | 2.3×10^-20^ | 0.42* | 0.31* | 0.75* |
| Eicosadienoic acid | 308.2715 | 7.37 | 3 | E | 4.1×10^-11^ | 0.43* | 0.25* | 0.59* |
|  |  |  |  | M | 5.1×10^-5^ | 0.41* | 0.33* | 0.81 |
|  |  |  |  | L | 6.2×10^-22^ | 0.45* | 0.29* | 0.66* |
| *Amino acids & derivatives* | | | | | | | | |
| L-Proline | 115.0633 | 0.74 | 1 | E | 6.2×10^-5^ | 0.85* | 0.89* | 1.05 |
|  |  |  |  | M | 3.6×10^-2^ | 0.93* | 0.93* | 1.00 |
|  |  |  |  | L | 3.3×10^-4^ | 0.90* | 0.92* | 1.02 |
| L-Tyrosine | 181.0738 | 1.29 | 1 | E | 1.5×10^-6^ | 0.74* | 0.71* | 0.95 |
|  |  |  |  | M | 4.8×10^-4^ | 0.78* | 0.69* | 0.88 |
|  |  |  |  | L | 3.3×10^-6^ | 0.83* | 0.72* | 0.87 |
| L-Leucine | 131.0946 | 1.50 | 1 | E | 7.0×10^-5^ | 0.78* | 0.65* | 0.83* |
|  |  |  |  | M | 3.3×10^-10^ | 0.74* | 0.60* | 0.82* |
|  |  |  |  | L | 3.6×10^-12^ | 0.77* | 0.61* | 0.80* |
| 3-Methyl-2-oxovaleric acid | 130.0629 | 2.77 | 1 | E | 2.8×10^-4^ | 0.70* | 0.45* | 0.65* |
|  |  |  |  | M | 2.5×10^-3^ | 0.65* | 0.51* | 0.78 |
|  |  |  |  | L | 1.2×10^-5^ | 0.69* | 0.42* | 0.61* |
| 4-Methyl-2-oxovaleric acid | 130.0629 | 2.93 | 1 | E | 3.7×10^-4^ | 0.63* | 0.44* | 0.69* |
|  |  |  |  | M | 1.5×10^-6^ | 0.56* | 0.45* | 0.81 |
|  |  |  |  | L | 4.2×10^-13^ | 0.59* | 0.30* | 0.52* |
| *Corticosteroids* | | | | | | | | |
| Cortisone | 360.1937 | 4.84 | 1 | E | 2.3×10^-3^ | 0.76* | 0.68* | 0.89 |
|  |  |  |  | M | 1.9×10^-3^ | 0.77* | 0.67* | 0.87 |
|  |  |  |  | L | 6.3×10^-5^ | 0.80* | 0.72* | 0.89 |
| Cortisol | 362.2093 | 5.00 | 1 | E | 1.4×10^-3^ | 0.79 | 0.51* | 0.64* |
|  |  |  |  | M | 2.9×10^-3^ | 0.71* | 0.66* | 0.93 |
|  |  |  |  | L | 2.3×10^-3^ | 0.77 | 0.65* | 0.84 |
| Tetrahydrocortisol | 366.2406 | 5.40 | 1 | E | 9.0×10^-3^ | 0.88 | 0.66* | 0.75 |
|  |  |  |  | M | 3.0×10^-2^ | 0.90 | 0.75* | 0.83 |
|  |  |  |  | L | 1.1×10^-4^ | 0.83* | 0.71* | 0.85* |
| *Other metabolites* | | | | | | | | |
| Hippuric acid | 179.0582 | 2.90 | 1 | E | 5.6×10^-5^ | 2.40* | 2.61* | 1.09 |
|  |  |  |  | M | 4.2×10^-3^ | 2.33* | 2.22* | 0.96 |
|  |  |  |  | L | 5.7×10^-9^ | 2.44* | 2.68* | 1.10 |

**Table 4.** Differential erythroid metabolites along the oral glucose tolerance test in children with late insulin peak, middle insulin peak, and early insulin peak. * Indicates significant differences according to the post-hoc Fisher LSD test (p < 0.05).

| **Metabolite** | **Mass (Da)** | **RT (min)** | **MSI level** | **group** | **p value** | **Fold changes** | | |
| --- | --- | --- | --- | --- | --- | --- | --- | --- |
|  |  |  |  |  |  | **60 vs. 0** | **120 vs. 0** | **120 vs. 60** |
| *Glycolytic intermediates* | | | | | | | | |
| D-Glucose 6-phosphate (or isomer) | 260.0297 | 0.69 | 3 | E | NS | 1.03 | 1.04 | 1.01 |
|  |  |  |  | M | NS | 1.02 | 1.02 | 1.00 |
|  |  |  |  | L | 3.5×10^-2^ | 1.06 | 1.20* | 1.13 |
| *Amino acids & derivatives* | | | | | | | | |
| L-Lysine | 146.1055 | 0.58 | 1 | E | 1.9×10^-2^ | 0.80* | 0.74* | 0.94 |
|  |  |  |  | M | NS | 0.85 | 0.84 | 0.99 |
|  |  |  |  | L | NS | 0.89 | 0.88 | 0.99 |
| L-Valine | 117.0790 | 0.87 | 1 | E | 3.9×10^-2^ | 0.70* | 0.70* | 1.00 |
|  |  |  |  | M | NS | 0.88 | 0.81 | 0.92 |
|  |  |  |  | L | NS | 0.83 | 0.79 | 0.95 |
| L-Methionine | 149.0510 | 0.96 | 1 | E | 3.4×10^-2^ | 0.81 | 0.70* | 0.87 |
|  |  |  |  | M | 2.4×10^-2^ | 0.89 | 0.74* | 0.84 |
|  |  |  |  | L | 2.3×10^-2^ | 0.86 | 0.77* | 0.90 |
| L-Tyrosine | 181.0738 | 1.30 | 1 | E | 4.0×10^-4^ | 0.82* | 0.81* | 0.98 |
|  |  |  |  | M | 3.5×10^-3^ | 0.88* | 0.82* | 0.93 |
|  |  |  |  | L | 5.8×10^-4^ | 0.88* | 0.83* | 0.94 |
| L-Leucine | 131.0946 | 1.48 | 1 | E | 8.7×10^-3^ | 0.79* | 0.75* | 0.95 |
|  |  |  |  | M | 2.3×10^-2^ | 0.83* | 0.78* | 0.94 |
|  |  |  |  | L | 2.7×10^-3^ | 0.83* | 0.77* | 0.93 |
| L-Phenylalanine | 165.0790 | 2.01 | 1 | E | 2.9×10^-2^ | 0.84* | 0.85* | 1.00 |
|  |  |  |  | M | 2.3×10^-2^ | 0.86* | 0.85* | 0.99 |
|  |  |  |  | L | 2.0×10^-2^ | 0.90* | 0.87* | 0.97 |
| L-Tryptophan | 204.0899 | 2.44 | 1 | E | 6.6×10^-3^ | 0.82* | 0.83* | 1.01 |
|  |  |  |  | M | 3.5×10^-3^ | 0.86* | 0.83* | 0.96 |
|  |  |  |  | L | 2.2×10^-4^ | 0.87* | 0.84* | 0.86 |
| 5-Hydroxytryptophan | 220.0848 | 1.62 | 1 | E | 2.8×10^-2^ | 0.86* | 0.89* | 1.03 |
|  |  |  |  | M | 3.4×10^-2^ | 0.93 | 0.87* | 0.94 |
|  |  |  |  | L | 2.5×10^-3^ | 0.91* | 0.88* | 0.97 |
| Ornithine | 132.0899 | 0.58 | 1 | E | 3.6×10^-3^ | 0.82* | 0.77* | 0.95 |
|  |  |  |  | M | 5.1×10^-3^ | 0.84* | 0.81* | 0.96 |
|  |  |  |  | L | 2.1×10^-3^ | 0.83* | 0.80* | 0.96 |
| N-Acetylmethionine | 191.616 | 2.69 | 1 | E | 3.6×10^-2^ | 0.83 | 0.66* | 0.80 |
|  |  |  |  | M | NS | 0.89 | 0.83 | 0.93 |
|  |  |  |  | L | NS | 0.82 | 0.78 | 0.94 |
| *Glutathione derivatives* | | | | | | | | |
| Cysteinyl-glycine | 178.0412 | 0.96 | 1 | E | NS | 0.98 | 0.96 | 0.97 |
|  |  |  |  | M | 3.1×10^-2^ | 0.87* | 0.80* | 0.92 |
|  |  |  |  | L | 1.1×10^-2^ | 0.88* | 0.90* | 1.03 |
| *Other metabolites* | | | | | | | | |
| Hippuric acid | 179.0582 | 2.91 | 1 | E | 1.0×10^-2^ | 1.29* | 1.18* | 0.91 |
|  |  |  |  | M | 1.4×10^-4^ | 1.26* | 1.18* | 0.94 |
|  |  |  |  | L | 1.2×10^-13^ | 1.47* | 1.39* | 0.94 |
